# Vericiguat Use in Patients with Heart Failure in Real-World Settings during the First Year after the Drug Authorization in Japan

**DOI:** 10.3390/jcm13113222

**Published:** 2024-05-30

**Authors:** Suguru Okami, Christoph Ohlmeier, Makiko Takeichi, Mireia Aguila, Katsiaryna Holl, Alexander Michel, Coralie Lecomte, Tomomi Ide

**Affiliations:** 1 Medical Affairs & Pharmacovigilance, Bayer Yakuhin Ltd., Breeze Tower, 2-4-9 Umeda, Kita-ku, Osaka 530-0001, Japan; 2 Integrated Evidence Generation & Business Innovation, Bayer AG, 13342 Berlin, Germany; 3 Aetion Inc., 5 Penn Plaza, New York, NY 10001, USA; 4 Integrated Evidence Generation & Business Innovation, Bayer Consumer Care AG, Peter Merian Straße 84, CH-4052 Basel, Switzerland; 5 Department of Cardiovascular Medicine, Faculty of Medical Sciences, Kyushu University, 3-1-1 Maidashi, Higashi-ku, Fukuoka 812-8582, Japan

**Keywords:** heart failure, vericiguat, titration, medical therapy, real-world

## Abstract

**Background:** Vericiguat was developed to treat patients with heart failure (HF). Currently, limited data are available to characterize vericiguat-treated patients in real-world clinical settings. **Methods:** This retrospective cohort study was done using a Japanese hospital administrative database to describe the use of vericiguat in patients with HF in real-world settings. Adult patients diagnosed with HF prescribed vericiguat between 1 July 2021 and 30 September 2022 were included. Patient characteristics at the initiation of vericiguat treatment, patterns of HF medication use, and vericiguat dose titrations were assessed within the first 90 days of treatment. **Results:** The study included 829 patients who were initiated on vericiguat therapy. The mean age was 75.5 years and 69.0% were male. Hypertension, coronary artery disease, and diabetes mellitus were present in 91.7, 71.3, and 60.1% of patients, respectively. Most patients had previously received HF medications, with high percentages using angiotensin-receptor blocker neprilysin inhibitors (ARNI; 43.9%) and sodium-glucose cotransporter-2 inhibitors (54.4%). During the first 90 days of vericiguat treatment, 65.8% of the patients were uptitrated from their starting dose, and 32.3% had reached the maximal daily dose. The median time to reach the maximal daily dose was 34 days. The multivariable model identified that initiating vericiguat treatment in an outpatient setting and using ARNI before initiating vericiguat treatment were factors significantly associated with reaching the maximal daily dose of vericiguat at any given time, whereas older age, chronic kidney disease, hyperkalemia, and anemia were not associated. **Conclusions:** These findings provide early insights into the use of vericiguat, which aid in optimizing the combinations and/or sequences of HF treatment incorporating vericiguat therapy.

## 1. Introduction

Heart failure (HF) is recognized as a global pandemic imposing a substantial socioeconomic burden [1,2,3]. HF is associated with high rates of morbidity and mortality. HF is punctuated by repeated worsening HF events, necessitating intravenous diuretics in an outpatient setting or hospitalization for HF [4,5]. Patients with worsening HF events have a high risk of subsequent HF readmission and mortality [6,7,8,9].

Vericiguat has been developed as a novel oral soluble guanylate cyclase stimulator for treating patients with chronic HF [10,11,12]. The VICTORIA randomized controlled trial evaluated the efficacy and safety of vericiguat among patients with HF with reduced ejection fraction (HFrEF) who experienced a worsening of HF [13]. Vericiguat was found to significantly reduce the risk of cardiovascular death or HF-related hospitalization when compared to the effects of placebo, with a hazard ratio of 0.90 (absolute event-rate reduction of 4.2 events per 100 patient-years) in patients with HFrEF at a high risk of a worsening HF event. Accordingly, the guidelines in the United States and Europe recommend considering vericiguat for the treatment of patients with symptomatic worsening of HFrEF (Class 2b) [14,15]. Vericiguat received marketing authorization in the United States and Europe in January 2021 and July 2021, respectively [16,17]. In Japan, vericiguat was approved in June 2021 for patients with chronic HF receiving standard treatments for chronic HF [18]. Randomized controlled trials typically apply stringent eligibility criteria to increase their internal validity, leading to the limited generalizability of trial findings to patients in clinical practice [19,20,21]. Currently, limited evidence is available to characterize patients treated with vericiguat in real-world clinical settings.

Herein, we aimed to report the characteristics and HF medication patterns of patients who initiated vericiguat treatment in real-world clinical settings upon the authorization of vericiguat in Japan. Furthermore, this study evaluated the dose titrations of vericiguat during the first 90 days after the initiation of treatment.

## 2. Methods

### 2.1. Study Design and Patient Selection

This retrospective cohort study used a nationwide hospital administrative database in Japan provided by Medical Data Vision Co., Ltd. (MDV; Tokyo, Japan). The MDV database comprises health insurance claims and clinical information collected from Diagnosis Procedure Combination (DPC) hospitals [22]. MDV is one of the largest datasets of hospital medical procedures and diagnoses according to the International Classification of Diseases 10th Revision codes, laboratory data, and prescriptions of inpatient and outpatient clinics. The dataset covers approximately 45 million patients who were treated at more than 400 hospitals across all geographical regions and age groups in Japan. The study period ranged from 1 January 2021 to 30 September 2022. Patients were identified based on a record of vericiguat prescriptions between 1 July 2021, corresponding to the date of marketing authorization in Japan (23 June 2021) and 30 September 2022 (identification period). The inclusion criteria were patients who initiated vericiguat treatment during the identification period, aged ≥ 18 years, without missing records of age and gender at the initiation of vericiguat treatment, and with at least 6 months of observability based on their medical records prior to initiating vericiguat treatment. The study did not set any specific exclusion criteria. Patients were followed up for 90 days from the date of vericiguat initiation (index date) to assess the utilization patterns of HF medications and the titration of vericiguat doseage.

### 2.2. Variables

Demographics and comedications were evaluated over a period of six months before the index date. Comorbidities and procedures were assessed across all available data before the index date. Records of prior worsening HF events, defined as the occurrence of either HF-related hospitalization or the prescription of intravenous diuretics in outpatient settings, were collected during the six months preceding the index date. The utilization patterns of HF medications, including beta-blockers, angiotensin-converting-enzyme inhibitors (ACEIs), angiotensin-receptor blockers (ARBs), angiotensin-receptor blocker neprilysin inhibitor (ARNI), mineralocorticoid receptor antagonists (MRAs), sodium-glucose cotransporter-2 inhibitors (SGLT2is), ivabradine, digoxin/digitoxin, and diuretics, were evaluated 90 days before and after the initiation of vericiguat treatment. Detailed definitions of these variables are summarized in Supplementary Table S1. The dosage patterns of renin-angiotensin-aldosterone system inhibitors (ACEI, ARB, ARNI, and MRA) and beta-blockers were summarized for each medication class and reported at baseline. The dosage patterns of these medication classes were categorized into three groups based on previous studies and Japanese guidelines: high dose, medium dose, and low dose [23,24,25]. Since SGLT2is do not require dose titrations, we did not include this class in the dosage patterns analysis. A list of the dosage categories for each drug is provided in Supplementary Table S2. We assessed the distribution of the first observed dose (“starting dose”) of vericiguat. Furthermore, the dosage patterns of vericiguat were described during the first 90 days after initiation, and were categorized into the following three groups: low daily dose (2.5 mg or below daily), intermediate daily dose (5 mg to <10 mg daily), and maximal daily dose (10 mg daily).

### 2.3. Statistical Analyses

Continuous variables are reported as the mean, standard deviation (SD), median, and interquartile range (IQR). Categorical variables are summarized as frequencies and percentages. No missing data were imputed. Patient characteristics and treatment patterns at the index date and during follow-up were descriptively summarized. The patterns and combinations of HF medication use were reported 90 days before and after the initiation of vericiguat treatment. This analysis required a minimum of seven days of treatment to be considered a valid treatment episode. Vericiguat dose titration patterns were assessed over 90 days after treatment initiation. The day-to-day titration patterns are graphically illustrated. The proportion of patients who were up- or downtitrated over 90 days after treatment initiation was calculated. Treatment discontinuation was identified based on a gap between successive prescription records of more than 30 days. The time required to reach the maximal daily dose of vericiguat was estimated. A multivariable Cox proportional hazards model was computed to assess the factors associated with reaching the vericiguat maximal daily dose at any given time over the 90 days of follow-up. The model included the time to reach the maximal daily dose during the 90 days of follow-up as the outcome and was adjusted for known predictors of HF medication uptitration [26,27], such as age, sex, comorbidities, and the use of HF medications and non-pharmacological therapies. The model computed hazard ratios (HRs) with 95% confidence intervals (CIs) for each variable included. Two-sided *p*-values were reported with a prespecified significance level of 0.05. The Wald test was applied to calculate the *p*-values of the HRs in the Cox proportional hazards model. Analyses for dose titrations over 90 days after the initiation of vericiguat treatment and the factors associated with reaching the maximal daily dose by the Cox regression model were performed in a subcohort of patients with at least 90 days of observability after the index date. The subgroup analyses were conducted in patients prescribed 2.5 mg of vericiguat as a starting dose. Statistical analyses were performed using the Aetion Evidence Platform (Aetion Substantiates). This study followed the STROBE (Strengthening the Reporting of Observational Studies in Epidemiology) statement [28], which is summarized in Supplementary Table S3. 

### 2.4. Ethics Approval

Given that this study utilized anonymized and de-identified secondary data, ethics committee approval was not needed. In Japan, ethical approval and informed consent do not apply to the use of de-identified secondary data in accordance with the Ethical Guidelines for Medical and Health Research Involving Human Subjects [29]. The use of de-identified data followed the local regulations including the Personal Information Protection Law. 

## 3. Results

### 3.1. Patient Characteristics

We identified 1086 patients who were prescribed vericiguat during the study period. After excluding 175 patients with <6 months of observability before the initiation of vericiguat treatment, one patient with missing age or sex records, and 81 patients who lacked follow-up records, 829 (76.3%) patients were included in the study. Of these patients, 520 (62.7%) initiated vericiguat treatment in hospital settings. A total of 738 (89.0%) patients were prescribed 2.5 mg of vericiguat as their first-observed dose (“starting dose”), and 75 (9.0%) patients were prescribed higher first-observed doses than 2.5 mg (Supplementary Table S4). Table 1 presents the characteristics of the overall study population and patients prescribed 2.5 mg of vericiguat as a starting dose, along with the characteristics of patients included in the VICTORIA trial [30,31,32]. The mean (±SD) age was 75.5 ± 11.8 years and 69.0% of patients were male. Hypertension, coronary artery disease, and diabetes mellitus were present in 91.7, 71.3, and 60.1% of patients, respectively. Overall, 34.7 and 16.6% of the patients had a previous history of myocardial infarction or stroke, respectively. In the overall study population, 268 patients (32.3%) had chronic kidney disease, and in patients prescribed 2.5 mg of vericiguat as a starting dose, 240 patients (32.5%) had chronic kidney disease. A total of 44.5% of patients had anemia at baseline, which was higher than the proportion reported in the VICTORIA trial. The use of ARNI (43.9%) or SGLT2i (54.4%) was observed more often in this study population than in the VICTORIA trial.

### 3.2. HF Medication Patterns before and after Vericiguat Initiation

**Figure 1:**
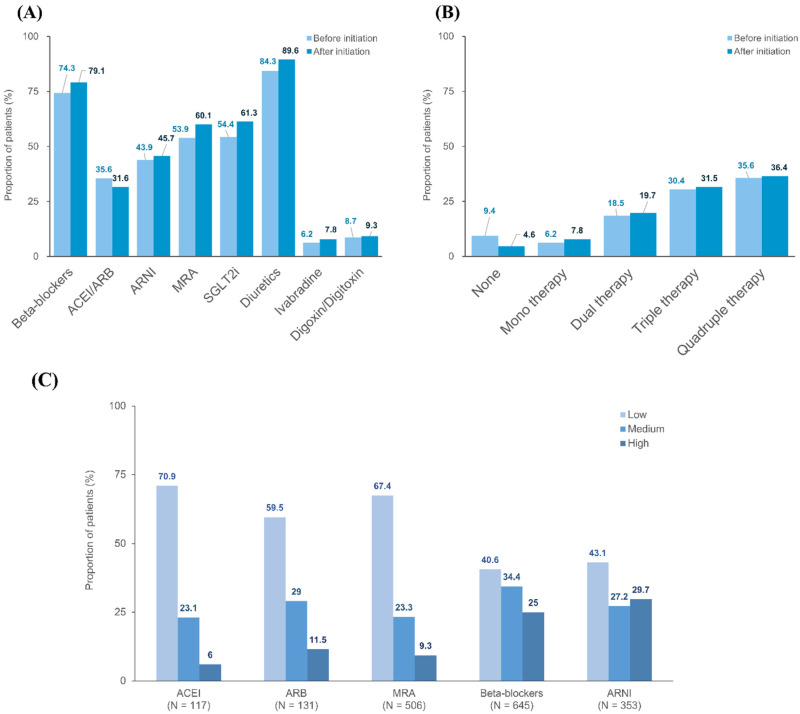
Utilization patterns of heart failure medications before and after initiating vericiguat treatment. Panel (**A**) shows the drug utilization patterns for each HF medication class 90 days before and after initiating vericiguat treatment, panel (**B**) shows the numbers of guideline-directed medical therapy for HF 90 days before and after initiating vericiguat treatment, and panel (**C**) shows the distributions of dosage category of each HF medication class at the initiation of vericiguat treatment. Analysis was performed in the overall study population (n = 829). Abbreviations: HF, heart failure; ACEI, angiotensin-converting-enzyme inhibitor; ARB, angiotensin-receptor blocker; MRA, mineralocorticoid receptor antagonist; ARNI, angiotensin-receptor blocker neprilysin inhibitor; and SGLT2i, sodium-glucose cotransporter-2 inhibitor.

Figure 1 presents the utilization patterns of HF medications before and after initiating vericiguat treatment in the study population. The use of HF medications increased after initiating vericiguat treatment, except for a slight decrease in ACEI/ARB use. Within 90 days before initiating vericiguat treatment, 51 (6.2%) and 78 (9.4%) patients received monotherapy or no guideline-directed medical therapy (GDMT; ACEI/ARB/ARNI, MRA, beta-blockers, or SGLT2i), respectively. The proportion of patients receiving dual, triple, and quadruple therapy of GDMT before initiating vericiguat treatment were 18.5, 30.4, and 35.6%, respectively (Figure 1; Supplementary Figure S1). The combined use of HF medications generally increased after initiating vericiguat treatment. At the initiation of vericiguat treatment, 60–70% of patients prescribed ACEI, ARB, or MRA were treated with a low dose. Higher percentages of medium- or high-dose categories were observed among patients prescribed beta-blockers or ARNI at the initiation of vericiguat treatment. The patterns of HF medication use before and after initiating vericiguat treatment in patients prescribed 2.5 mg of vericiguat as a starting dose are shown in Supplementary Figure S2. 

### 3.3. Vericiguat Dose Titration over 90 Days after Initiation

**Figure 2:**
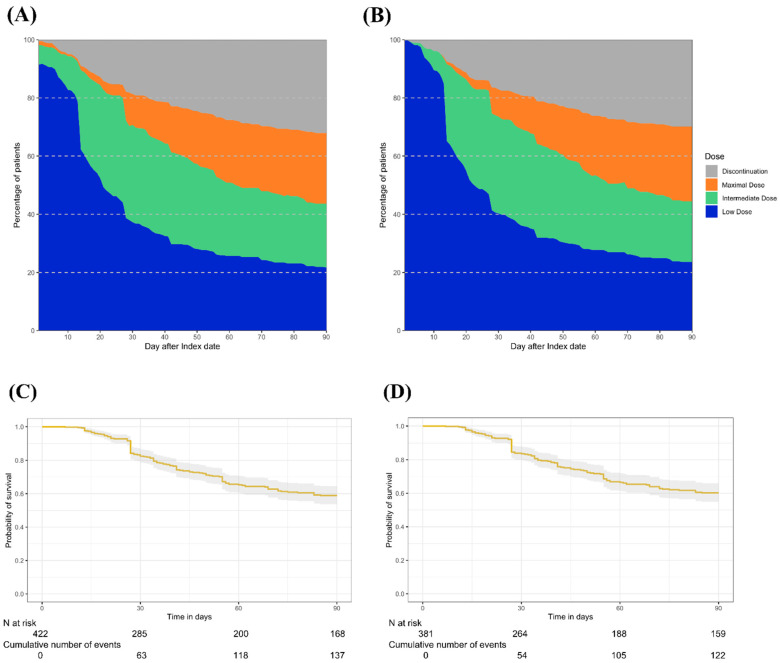
Dose titration of vericiguat over 90 days after initiation of treatment. Panel (**A**) shows the day-to-day titration patterns of vericiguat in all patients and panel (**B**) shows those in patients with 2.5 mg of vericiguat as a starting dose. Panel (**C**) shows the Kaplan Meier curve of reaching vericiguat maximal dose in all patients and panel (**D**) shows that in patients with 2.5 mg of vericiguat as a starting dose. The dosage patterns of vericiguat were categorized into the following three groups: low daily dose (≤2.5 mg daily), intermediate daily dose (5 mg to <10 mg daily), and maximal daily dose (10 mg daily). Treatment discontinuation was identified by the gap between successive prescription records for >30 days.

The patterns of vericiguat dose titration were assessed in a subset of patients who underwent at least 90 days of follow-up (n = 424). The characteristics of the patients in this subset were similar to those patients comprising the overall study population (Supplementary Table S5). Figure 2 A and B depict the day-to-day patterns of vericiguat dose titrations over 90 days after the initiation of treatment. The proportion of patients receiving intermediate and maximal daily doses increased steadily over 90 days, with marked increases observed on days 14 and 28. The proportion of patients who experienced uptitration at 30, 60, and 90 days were 55.0, 63.7, and 65.8%, respectively (Table 2). The Kaplan–Meier curves, showing the time to reach the vericiguat maximal daily dose, indicate that approximately 40% of patients reached the maximal daily dose on day 90 (Figure 2C,D). The median (IQR) time to reach the maximal daily dose was 34 (27–54.5) days. Table 3 shows the factors associated with reaching the maximal daily dose of vericiguat at any given time over 90 days following treatment initiation. The multivariable Cox regression model identified vericiguat treatment initiation in the outpatient setting (HR [95% CI]: 1.79 [1.27–2.52]), and the use of ARNI before initiating vericiguat treatment (HR [95% CI]: 1.56 [1.08–2.25]) was significantly associated with reaching the maximal daily dose of vericiguat at any given time over 90 days following treatment initiation.

## 4. Discussion

Herein, we report current information on vericiguat use in real-world clinical settings just after drug authorization in Japan, including patient characteristics, patterns of HF medication use, and dose titrations of vericiguat during the first 90 days after initiation of treatment.

The patients in this current study were older and comprised a lower proportion of males than in the VICTORIA trial. Similar to the VICTORIA trial participants, the patients included in this present study had multiple comorbidities, exhibiting high percentages of cardiovascular and metabolic complications, including hypertension, coronary artery disease, diabetes mellitus, and a history of myocardial infarction. Several differences were observed compared to the VICTORIA trial, including the percentage of patients with anemia and those treated with an implantable cardioverter defibrillator. The high percentage of patients with anemia in this present study is consistent with the previously reported prevalence of anemia among patients with HF [33,34], suggesting its importance as a commonly encountered complication associated with poor clinical status or worse outcomes [35]. We found that vericiguat was prescribed even in patients who did not have worsening HF events within 6 months prior to initiating vericiguat treatment, suggesting that vericiguat was used in patients with deteriorating conditions, but not necessarily in those experiencing a recent worsening HF event, in real-world settings. 

The use of HF medications that more recently became available in Japan such as ARNI (43.9% in the current study vs. 14.5% in the VICTORIA trial) and SGLT2i (54.4% vs. 2.7%) was more frequently observed in the current study than that in the VICTORIA trial, whereas ACEI or ARB (35.6% vs. 73.5%), beta-blockers (74.3% vs. 93.1%), and MRA (53.9% vs. 70.4%) were less frequently observed than in the VICTORIA trial. These differences likely reflect the increasing availability of treatment options for patients with HFrEF during the last couple of years. Overall treatment intensification was observed in the study population which vericiguat was part of. The use of comedications observed in our study was comparable to that in a registry-based study involving patients with similar inclusion/exclusion criteria as in the VICTORIA trial, which points towards the generalizability of results from the pivotal trial to the wider Japanese HFrEF population [36].

Although our study results suggest that a considerable number of patients were treated with contemporary medical management using newer types of HF medications (ARNI or SGLT2i), relatively few patients received all four recommended GDMT prior to initiating vericiguat treatment (35.6%). Thus, vericiguat seems to be used earlier in the treatment cascade than recommended [14], which might point towards challenges in implementing GDMT in real-world clinical settings. These challenges may be due to the intolerability to GDMT and the risk of adverse events such as hypotension and hyperkalemia [37,38,39].

The recently published clinical consensus statement by the Heart Failure Association of the European Society of Cardiology suggests that preventing HF worsening is a major target for the current treatment of HF [40]. This is reinforced by Greene et al. [41], who recommend the initiation of GDMT simultaneously or in a rapid sequence and the addition of vericiguat as important components of prevention therapy. Dose escalation of oral medical therapies to the maximally tolerated or target doses is recommended as a subsequent step to further magnify the benefits of these therapies. Discontinuation episodes were observed in 31.1% of the overall study population and 29.1% of patients with 2.5 mg of vericiguat as a starting dose. In the EVOLUTION HF study, discontinuation episodes of GDMT for HF within 12 months of treatment initiation were observed in 24.4–68.9% of the study population [42]. Given that most of the discontinuation episodes related to HF medications were observed within the first 90 days after the treatment initiation across studies [43,44], our study suggests vericiguat therapy may be similarly persistent to other GDMT in real-world clinical settings. Nakamura et al. reported their initial experience with vericiguat therapy prescribed to patients [45] and found that 4 of the 28 patients discontinued vericiguat owing to symptomatic hypotension or impaired swallowing function. Among the 21 patients who continued vericiguat therapy for >4 months, 12 (57%) and 5 (24%) were uptitrated to 10 and 5 mg daily, respectively, which is in line with or slightly higher than that observed in the current study. 

ARNI use and vericiguat treatment initiation in outpatient settings were positively associated with reaching the maximal daily dose of vericiguat at any given time over 90 days of treatment initiation. It can be speculated that patients who continued to be on ARNI were more likely to be tolerant to the addition and uptitration of vericiguat. It should also be noted that factors usually considered to impede the continuation of HF medications, such as older age, presence of hyperkalemia, chronic kidney disease, and anemia [38,39], were not associated with reaching the maximal daily dose of vericiguat. This suggests that vericiguat can successfully be uptitrated, even in these challenging subpopulations, potentially pointing towards a favorable safety profile in routine care settings. The time until uptitration was well in line with recommendations, which is another finding to support the favorable tolerability of vericiguat in routine care settings.

This current study had several limitations. First, limited laboratory data were available in the MDV; therefore, we did not assess the trajectories of brain natriuretic peptide levels before and after vericiguat administration. Echocardiography, New York Heart Association class, and blood pressure data were unavailable for the dataset, precluding the analysis based on these parameters. Previous research showed that some key echocardiographic data, such as right ventricular function (e.g., tricuspid annulus plane systolic excursion), were useful in predicting the maximum dose tolerability of ARNI [46]. Second, the relatively short follow-up duration may have limited the generalizability of the findings to a longer period, which needs to be addressed in future studies. Third, only structured information was recorded in the MDV; therefore, qualitative information, such as the reasons for treatment initiation/discontinuation, could not be assessed. The causes for hospitalization could not be assessed; therefore, we could not fully exclude the possibility of underreporting HF-related hospitalization before the initiation of vericiguat treatment. Likewise, the daily dose of vericiguat was calculated based on the prescription record, since the information on the dose selected by physicians was not recorded. Notably, patients could not be followed up across different institutions in the MDV, which could have limited the duration of patient follow-up. The analysis of the dose titration of vericiguat included only a subset of patients with at least 90 days of follow-up; therefore, the results were only applicable to those surviving the first 90 days and continued follow-up.

Herein, we present initial evidence on vericiguat use in real-world clinical settings during the first year following drug authorization in Japan. Patients were largely comparable to the VICTORIA trial population indicating the generalizability of trial results to the wider population of vericiguat users in real-world settings. Two-thirds of vericiguat patients were uptitrated, with one-third reaching the maximal daily dose. Factors usually considered to impede uptitration, such as older age, chronic kidney disease, presence of hyperkalemia, and anemia, were not associated with reaching the maximal daily dose at any given time over 90 days following treatment initiation, pointing towards good tolerability of vericiguat also in routine care settings. Overall, the initiation of vericiguat treatment seemed to occur earlier in the HF treatment sequences than recommended, potentially reflecting the challenges regarding initiation/uptitration of GDMT. Collectively, these findings provide early insights into the use of vericiguat in real-world clinical settings, which will aid in optimizing combinations and/or sequences of HF treatments incorporating vericiguat therapy.

## Figures and Tables

**Table 1:** Baseline characteristics of all patients and patients who received 2.5 mg of vericiguat as a starting dose, along with the characteristics of patients included in the VICTORIA trial. Abbreviations: SD, standard deviation; IQR, interquartile range; ACEI, angiotensin-converting enzyme inhibitor; ARB, angiotensin-receptor blocker; MRA, mineralocorticoid receptor antagonist; ARNI, angiotensin-receptor blocker neprilysin inhibitor. * Calculated in patients who had prior worsening heart failure. ** Used during a period of 90 days before the index date. *** Reported in 5048 patients who had coronary artery disease status data available in the VICTORIA trial [31]. **** Only the percentage of patients was available in the reference [32].

	Overall (N = 829)	Patients with 2.5 mg as a Starting Dose (N = 738)	VICTORIA [30,31,32] (N = 5050)
Age (years)			
Mean ± SD	75.5 ± 11.8	75.5 ± 11.7	67.3 ± 12.2
Median (IQR)	77 (69–84)	77 (69–84)	69 (60–76)
Gender, male, n (%)	572 (69.0)	510 (69.1)	3842 (76.1)
Body mass index, kg/m^2^			
Median (IQR)	22.1 (19.7–24.9)	22.1 (19.8–24.9)	26.8 (23.7–30.9)
Missing, n (%)	237 (28.6)	204 (27.6)	46 (0.9)
Prior worsening heart failure event, n (%)	271 (32.7)	248 (33.6)	5050 (100)
Hospitalization for heart failure	198 (23.9)	184 (24.9)	4237 (83.9)
Outpatient intravenous diuretics prescription	103 (12.4)	93 (12.6)	813 (16.1)
Time from worsening heart failure event (days) *			
Mean ± SD	41.2 ± 47.0	40.7 ± 46.5	–
Median (IQR)	20 (6–66)	20 (6–65)	–
Comorbidity, n (%)			
Hypertension	760 (91.7)	681 (92.3)	3993 (79.1)
Coronary artery disease	591 (71.3)	529 (71.7)	2942 (58.3)
Diabetes mellitus	498 (60.1)	446 (60.4)	2377 (47.1)
Myocardial infarction	288 (34.7)	262 (35.5)	2121 (42.0) ***
Atrial fibrillation	272 (32.8)	240 (32.5)	2269 (45.0)
Stroke	138 (16.6)	120 (16.3)	579 (11.5)
Anemia	369 (44.5)	334 (45.3)	1053 (20.9)
Cardiovascular procedure, n (%)			
Biventricular pacemaker	91 (11.0)	77 (10.4)	739 (14.7)
Implantable cardioverter defibrillator	102 (12.3)	86 (11.7)	1399 (27.8)
HF treatments before the index date **, n (%)			
ACEI or ARB	295 (35.6)	269 (36.4)	3704 (73.5)
Beta-blockers	616 (74.3)	562 (76.2)	4691 (93.1)
MRA	447 (53.9)	411 (55.7)	3548 (70.4)
ARNI	364 (43.9)	335 (45.4)	731 (14.5)
SGLT2i	451 (54.4)	418 (56.6)	2.7% ****

**Table 2:** Patterns of dose titration of vericiguat over 90 days after treatment initiation. Abbreviations: SD, standard deviation; IQR, interquartile range.

	All Patients with ≥90 Days Follow-Up (N = 424)	Patients with 2.5 mg as a Starting Dose (N = 382)
Patients with uptitration, n (%)		
At 30 days	233 (55.0)	225 (58.9)
At 60 days	270 (63.7)	258 (67.5)
At 90 days	279 (65.8)	266 (69.6)
Patients reached the maximal daily dose, n (%)	137 (32.3)	122 (31.9)
Time to reach the maximal daily dose, days		
Mean ± SD	38.4 ± 18.9	38.7 ± 18.9
Median (IQR)	34 (27–54.5)	34 (27–55)
Patients downtitrated, n (%)	10 (2.4)	0 (0)
Patients discontinued treatment, n (%)	132 (31.1)	111 (29.1)

**Table 3:** Results of univariable and multivariable Cox proportional hazard models for the factors associated with reaching the maximal daily dose of vericiguat at any given time over 90 days after treatment initiation. Abbreviations: SD, standard deviation; HR, hazard ratio; CI, confidence interval; ACEI, angiotensin-converting enzyme inhibitor; ARB, angiotensin-receptor blocker; MRA, mineralocor-ticoid receptor antagonist; ARNI, angiotensin-receptor blocker neprilysin inhibitor; SGLT2i, sodium-glucose cotransporter-2 inhibitor; GDMT, guideline-directed medical therapy; HF, heart failure. * Assessed using all available data prior to the index date. ** Assessed during 183 days prior to the index date (not included index date). *** GDMT included beta-blockers, ACEI, ARB, ARNI, MRA, and SGLT2i.

	Univariate HR		Multivariable HR	
	HR (95% CI)	*p*-Value	HR (95% CI)	*p*-Value
*Age, years*				
<75	Reference	Reference	Reference	Reference
≥75	0.94 (0.67–1.31)	0.701	0.99 (0.70–1.40)	0.965
*Gender*				
Male	Reference	Reference	Reference	Reference
Female	0.68 (0.46–1.00)	0.052	0.70 (0.47–1.05)	0.082
*Vericiguat initiation setting*				
Inpatient	Reference	Reference	Reference	Reference
Outpatient	1.82 (1.29–2.55)	0.001	1.79 (1.27–2.52)	0.001
*Vericiguat first observed dose, mg*				
≤2.5	Reference	Reference	Reference	Reference
>2.5	1.22 (0.70–2.13)	0.475	–	–
Comorbidities *				
CKD	0.97 (0.68–1.40)	0.883	–	–
Hyperkalemia	0.92 (0.62–1.37)	0.691	–	–
Anemia	0.81 (0.57–1.14)	0.225	–	–
Atrial fibrillation	1.19 (0.84–1.69)	0.335	–	–
Hypertension	3.43 (1.09–10.78)	0.035	3.15 (1.00–9.91)	0.050
Device and procedure history *				
Biventricular pacemaker	0.95 (0.57–1.59)	0.857	–	–
Implantable cardioverter defibrillator	0.74 (0.44–1.22)	0.238	–	–
HF medications **				
ACEI or ARB	1.01 (0.72–1.41)	0.958	–	–
Beta-blockers	1.61 (1.01–2.57)	0.043	1.27 (0.78–2.05)	0.336
MRA	1.02 (0.72–1.44)	0.915	–	–
ARNI	1.80 (1.27–2.54)	0.001	1.56 (1.08–2.25)	0.018
SGLT2i	1.50 (1.06–2.13)	0.024	1.15 (0.79–1.68)	0.467
Inotrope	0.89 (0.62–1.26)	0.498	–	–
Number of GDMT ***				
No use	Reference	Reference	Reference	Reference
Mono therapy	0.16 (0.02–1.27)	0.083	–	–
Dual therapy	1.36 (0.58–3.17)	0.483	–	–
Triple therapy	1.39 (0.63–3.08)	0.417	–	–
Quadruple therapy	1.63 (0.75–3.57)	0.218	–	–

## Data Availability

The data included in this manuscript were used under contract with the provider (Medical Data Vision) and cannot be freely distributed by the authors. All necessary data required to interpret and conclude the findings of this study were included in the main text and supplementary materials.

## Supplementary Materials

The following supporting information can be downloaded at https://www.mdpi.com/article/10.3390/jcm13113222/s1, Table S1: List of variables used to assess eligibility, comorbidities, and comedications in the study [47]. Table S2: List of dosage categories for each heart failure medication. Table S3: STROBE (Strengthening the Reporting of Observational Studies in Epidemiology) Checklist of the study [28]. Table S4: First observed dose (“starting dose”) of vericiguat in overall patients and by initiation settings. Table S5: Baseline characteristics of all patients and patients in the subset with ≥90 days of follow-up. Table S6: Results of univariable and multivariable Cox proportional hazard models for the factors associated with reaching the maximal daily dose of vericiguat at any given time over 90 days of treatment initiation in patients with vericiguat 2.5 mg as a starting dose. Figure S1: Patterns of guideline-directed medical therapy combinations for heart failure before and after vericiguat initiation. Figure S2: Utilization patterns of heart failure medications before and after vericiguat initiation in patients with vericiguat 2.5 mg as a starting dose.

## Abbreviations

ACEI = angiotensin-converting-enzyme inhibitor; ARB = angiotensin-receptor blocker; ARNI = angiotensin-receptor blocker neprilysin inhibitor; CI = confidence interval; DPC = diagnosis procedure combination; GDMT = guideline-directed medical therapy; HF = heart failure; HFrEF = heart failure with reduced ejection fraction; HR = hazard ratio; IQR = inter-quartile range; MDV = Medical Data Vision database; MRA = mineralocorticoid receptor antagonist; SD = standard deviation; SGLT2i = sodium-glucose cotransporter-2 inhibitor.
