# Supplementary material for: Vericiguat Use in Patients with Heart Failure in Real-World Settings during the First Year after the Drug Authorization in Japan
Source: J Clin Med. 2024 May 30;13(11):3222. doi: 10.3390/jcm13113222 (PMC11172519; doi:10.3390/jcm13113222)
Supplement: Supplementary file 1 [file jcm-13-03222-s001.zip › jcm-2931871-supplementary.pdf]

APPENDIX

Table S1. List of variables used to assess eligibility, comorbidities, and comedications in the study..2

Table S2. List of dosage categories for each heart failure medication. 5

Table S3. STROBE (Strengthening the Reporting of Observational Studies in Epidemiology) Checklist of the study..6

Table S4. First observed dose ("starting dose") of vericiguat in overall patients and by initiation settings..8

Table S5. Baseline characteristics of all patients and patients in the subset with ≥90 days of follow-up 9

Table S6. Results of univariable and multivariable Cox proportional hazard models for the factors associated with reaching the maximal daily dose of vericiguat at any given time over 90 days of treatment initiation in patients with vericiguat 2.5 mg as a starting dose 10

Fig. S1. Patterns of guideline-directed medical therapy combinations for heart failure before and after vericiguat initiation...11

Fig. S2. Utilization patterns of heart failure medications before and after vericiguat initiation in patients with vericiguat 2.5 mg as a starting dose..12

References..13

Supplementary tables and figures

Table S1. List of variables used to assess eligibility, comorbidities, and comedications in the study. Abbreviations: ACEI, angiotensin-converting-enzyme inhibitor; ARB, angiotensin-receptor blocker; ARNI, angiotensin-receptor blocker neprilysin inhibitor; BB, beta-blocker; MRA, mineralocorticoid receptor antagonist; ICD-10, International Classification of Disease, 10th Revision; eGFR, estimated glomerular filtration rate; EphMRA, European Pharmaceutical Market Research Association; ATC, Anatomical Therapeutic Chemical.

**Variables**	**Definition**
Variables to assess the eligibility	
Heart failure	Any inpatient or outpatient record with a diagnosis (any position) of heart failure (ICD-10: I50, I11.0)
Vericiguat	Any inpatient and outpatient claims codes of vericiguat (622871001, 622871101, 622871201) or generic drug name of vericiguat.
Comorbidities	
Hypertension	Any inpatient or outpatient record with a diagnosis (any position) of hypertension (ICD-10: I10–13, I15, I16.9, I67.4, O11, O16)
Chronic kidney disease	Any inpatient or outpatient diagnosis (any position) or healthcare claims codes of chronic kidney disease. (ICD-10: I12.0, I13.2, N17–19, Z94.0, Z94.0, Z99.2; claims code, 140007710, 140007910, 140008170, 140033770, 140036710, 140051010, 140051110, 140052570, 140052810, 140052970, 140055970, 140057810, 140057910, 140058010, 140058110, 140058210, 140058310, 140058410, 140058510, 140058610, 140058770, 140058870, 140058970, 140059070, 140059170, 140059310, 140059410, 140059510, 140060210, 140060310, 140060410, 140060510, 140060610, 140060710, 140060810, 140060910, 140061010, 140062770, 140062870, 150196310, 150196410, 150196570, 150324810, 150338610, 150419370, 150420970, 150421070; procedure code, K780, K780-2, J038, J038-2)
Coronary artery disease	Any inpatient or outpatient record with a diagnosis (any position) of coronary artery disease (ICD-10: I20–21, I24–25)
Atrial fibrillation	Any inpatient or outpatient record with a diagnosis (any position) of atrial fibrillation (ICD-10: I48.0, I48.1, I48.2)
Diabetes mellitus	Any inpatient or outpatient record with a diagnosis (any position) of diabetes mellitus (ICD-10: E8–11, E13)
Stroke	Any inpatient or outpatient record with a diagnosis (any position) of ischemic stroke or intracerebral hemorrhage (ICD-10: I60–63)
Myocardial infarction	Any inpatient or outpatient record with a diagnosis (any position) of myocardial infarction (ICD-10: I21–23, I24.0, I25.2, I25.6)
Anemia	Any inpatient or outpatient record with a diagnosis (any position) of anemia (ICD-10: D50.9, D53, D59, D61, D63, D64)
Hyperkalemia	Any inpatient or outpatient record with a diagnosis (any position) of hyperkalemia (ICD-10: E87.5)
Cardiovascular procedures	

Biventricular pacemaker	Procedure codes (K598, K598-2, K599-3, K599-4) or claims codes for biventricular pacemakers (150303210, 150415210, 150322210, 150415410, 150415110, 150415310, 150336910, 150337010, 150415910, 150416110, 150415810, 150416010)
Implantable cardioverter defibrillator	Procedure codes (K599, K599-2, K599-3, K599-4) or claims codes of implantable cardioverter defibrillator (150387410, 150336910, 150275310, 150275210, 150337010, 150415710, 150415910, 150383250, 150416110, 150415810, 150415610, 150415510, 150416010)
Worsening event	
Worsening heart failure event	Start of heart failure hospitalization (inpatient claim with a "confirmed" diagnosis code of heart failure) or at least one prescription of intravenous diuretics in the outpatient setting (identified by its General Drug Name in English, by its Health Claim Codes, or by EphMRA ATC codes) [1] For patients initiated vericiguat in-hospital, the ascertainment of heart failure hospitalization will be performed from admission to discharge of index hospitalization.
Heart failure hospitalization	Inpatient claim with a "confirmed" diagnosis code of heart failure. For patients initiated on vericiguat in the hospital, the ascertainment of heart failure hospitalization will be performed from admission to discharge of index hospitalization.
Outpatient intravenous diuretics	Prescription of intravenous diuretics in an outpatient setting.
Heart failure medication	
ACEI	Any inpatient or outpatient record with EphMRA ATC code of ACEI (C09A0) or generic drug names of ACEI.
ARB	Any inpatient or outpatient record with EphMRA ATC code of ARB (C09C0, C09D1, C09D3) or generic drug names of ARB.
Beta-blocker	Any inpatient and outpatient claims codes of beta-blockers (610453017, 610453018, 610453097, 610453122, 610453123, 610453124, 610453125, 610453127, 610462039, 610462040, 610463009, 610463010, 610463013, 612120266, 612120267, 612140702, 612140703, 620000005, 620000006, 620001874, 620001875, 620002018, 620002708, 620002709, 620256101, 620256201, 621399302, 621399401, 621399501, 621399602, 621399902, 621400001, 621400301, 621400302, 621400401, 621400502, 621400601, 621400901, 621470101, 621470201, 621470301, 621470401, 621520501, 621520504, 621520601, 621520604, 621573401, 621573402, 621681901, 621682001, 622049001, 622049101, 622060401, 622060402, 622064802, 622068001, 622068002, 622095501, 622095601, 622206801, 622206901, 622267901, 622271001, 622284601, 622289401, 622289402, 622296701, 622296801, 622312800, 622339301, 622355501, 622355601, 622380901, 622381001, 622479201, 622479301, 622481401, 622481501, 622490801, 622490901, 622494301, 622494302, 622494401, 622496101, 622496103, 622496201, 622496203, 622500301, 622500401, 622504201, 622504301, 622591001, 622591101, 622591201, 622644401, 622644501, 622644601, 622644701, 622719200, 622719300,

	622725200, 622725300, 622725400, 622872701, 622872801, 622872901, 622873001, 622875901, 622876001, 622876101) or generic drug names of beta-blockers.
MRA	Any inpatient or outpatient record with EphMRA ATC code of MRA (C03A1) or claims code of Eplerenone (610443011, 610443012, 620000139) or generic drug names of MRA.
ARNI	Any inpatient or outpatient record with EphMRA ATC code of Sacubitril/valsartan (C09D9) or generic drug names of sacubitril/ valsartan.
SGLT2i	Any inpatient or outpatient record with EphMRA ATC code of SGLT2i (A10P1) or generic drug names of SGLT2i.
Diuretics	Any inpatient or outpatient record with EphMRA ATC code of Diuretics (C03A2, C03A3, C03A7) or generic drug names of Diuretics.
Digoxin/digitoxin	Any inpatient or outpatient record with EphMRA ATC code C01A1 or generic drug names of digoxin/digitoxin.
Ivabradine	Any inpatient or outpatient record with claims codes of ivabradine (622698601, 622698701, 622698801) or generic drug name of ivabradine.
Inotrope	Any inpatient or outpatient record with EphMRA ATC code C01C1, C01F0, or generic drug names of inotrope.

Table S2. List of dosage categories for each heart failure medication. Abbreviations: ACEI, angiotensin-converting-enzyme inhibitor; ARB, angiotensin-receptor blocker; ARNI, angiotensin-receptor blocker neprilysin inhibitor; BB, beta-blocker; HF, heart failure; MRA, mineralocorticoid receptor antagonist.

**Drug**	**Target Maximum Dose**	**High Dose**	**Medium Dose**	**Low Dose**
ACEI				
Captopril	150 mg daily	150 mg daily	105 mg daily	45 mg daily
Enalapril	10 mg daily	10 mg daily	7 mg daily	3 mg daily
Lisinopril	20 (HF=10) mg daily	10 mg daily	7 mg daily	3 mg daily
Trandolapril	2 mg daily	2 mg daily	1.4 mg daily	0.6 mg daily
Alacepril	100 mg daily	100 mg daily	70 mg daily	30 mg daily
Benazepril	10 mg daily	10 mg daily	7 mg daily	3 mg daily
Cilazapril	2 mg daily	2 mg daily	1.4 mg daily	0.6 mg daily
Derapril	120 mg daily	120 mg daily	84 mg daily	36 mg daily
Imidapril	10 mg daily	10 mg daily	7 mg daily	3 mg daily
Perindopril	8 mg daily	8 mg daily	5.6 mg daily	2.4 mg daily
Quinapril	20 mg daily	20 mg daily	14 mg daily	6 mg daily
Temocapril	4 mg daily	4 mg daily	2.8 mg daily	1.2 mg daily
ARB				
Candesartan	12 (HF=8) mg daily	8 mg daily	5.6 mg daily	2.4 mg daily
Losartan	100 mg daily	100 mg daily	70 mg daily	30 mg daily
Valsartan	160 mg daily	160 mg daily	112 mg daily	48 mg daily
Azilsartan	40 mg daily	40 mg daily	28 mg daily	12 mg daily
Irbesartan	200 mg daily	200 mg daily	140 mg daily	60 mg daily
Olmesartan	40 mg daily	40 mg daily	28 mg daily	12 mg daily
Telmisartan	80 mg daily	80 mg daily	56 mg daily	24 mg daily
ARNI				
Sacubitril/Valsartan	97 mg sacubitril and 103 mg valsartan twice daily	97 mg sacubitril and 103 mg valsartan twice daily	49 mg sacubitril and 51 mg valsartan twice daily	24 mg sacubitril and 26 mg valsartan twice daily
BB				
Bisoprolol	1.25–5 mg daily	2.5-5 mg daily	1.25–2.5 mg daily	0.625-1.25 mg daily
Carvedilol	5–20 mg twice daily	10–20 mg twice daily	5–10 mg twice daily	2.5–5 mg daily
MRA				
Spironolactone	100 mg daily	100 mg daily	70 mg daily	30 mg daily
Eplerenone	100 (HF = 50) mg daily	50 mg daily	35 mg daily	15 mg daily

Table S3. STROBE (Strengthening the Reporting of Observational Studies in Epidemiology) Checklist of the study [2].

	Item No.	Recommendation	Page No.
**Title and abstract**	1	(*a*) Indicate the study design with a commonly used term in the title or abstract	2
		(*b*) Provide in the abstract an informative and balanced summary of what was done and what was found	2
**Introduction**			
Background/ rationale	2	Explain the scientific background and rationale for the investigation being reported	5
Objectives	3	State the specific objectives, including any prespecified hypotheses	5
**Methods**			
Study design	4	Present key elements of study design early in the paper	6
Setting	5	Describe the setting, locations, and relevant dates, including periods of recruitment, exposure, follow-up, and data collection	6
Participants	6	(*a*) *Cohort study*—Specify the eligibility criteria and the sources and methods of selection of participants. Describe methods of follow-up	6
		(*b*) For matched studies, provide matching criteria and the number of exposed and unexposed	N/A
Variables	7	Clearly define all outcomes, exposures, predictors, potential confounders, and effect modifiers. Give diagnostic criteria, if applicable	6
Data sources/ measurement	8	For each variable of interest, give sources of data and details of methods of assessment (measurement). Describe the comparability of assessment methods if there is more than one group	6–7
Bias	9	Describe any efforts to address potential sources of bias	7
Study size	10	Explain how the study size was arrived at	8
Quantitative variables	11	Explain how quantitative variables were handled in the analyses. If applicable, describe which groupings were chosen and why	7
Statistical methods	12	(*a*) Describe all statistical methods, including those used to control for confounding	7
		(*b*) Describe any methods used to examine subgroups and interactions	7
		(*c*) Explain how missing data were addressed	7
		(*d*) If applicable, explain how the loss to follow-up was addressed	N/A
		(e) Describe any sensitivity analyses	N/A

Continued on the next page

**Results**			
Participants	13	(a) Report numbers of individuals at each stage of study—e.g., numbers potentially eligible, examined for eligibility, confirmed eligible, included in the study, completing follow-up, and analyzed	8
		(b) Give reasons for non-participation at each stage	8
		(c) Consider the use of a flow diagram	N/A
Descriptive data	14	(a) Give characteristics of study participants (e.g., demographic, clinical, social) and information on exposures and potential confounders	8
		(b) Indicate the number of participants with missing data for each variable of interest	19
		(c) Summarize follow-up time (e.g., average and total amount)	N/A
Outcome data	15	Report numbers of outcome events or summary measures over time	20
Main results	16	(*a*) Give unadjusted estimates and, if applicable, confounder-adjusted estimates and their precision (e.g., 95% confidence interval). Make clear which confounders were adjusted for and why they were included	21
		(*b*) Report category boundaries when continuous variables were categorized	21
		(*c*) If relevant, consider translating estimates of relative risk into absolute risk for a meaningful time period	N/A
Other analyses	17	Report other analyses performed—e.g., analyses of subgroups and interactions and sensitivity analyses	8, 20
**Discussion**			
Key results	18	Summarize key results with reference to study objectives	10
Limitations	19	Discuss the limitations of the study, taking into account sources of potential bias or imprecision. Discuss both the direction and magnitude of any potential bias	12
Interpretation	20	Give a cautious overall interpretation of results considering objectives, limitations, the multiplicity of analyses, results from similar studies, and other relevant evidence	10,11
Generalizability	21	Discuss the generalizability (external validity) of the study results	12
**Other information**			
Funding	22	Give the source of funding and the role of the funders for the present study and, if applicable, for the original study on which the present article is based	16

Table S4. First observed dose ("starting dose") of vericiguat in overall patients and by initiation settings. Abbreviations: SD, standard deviation; and IQR, interquartile range.

	**Overall (N = 829)**	**In-hospital (N = 520)**	**Outpatient (N = 309)**
First observed daily dose of vericiguat at initiation, mg			
Mean ± SD	2.84 (1.62)	2.82 (1.87)	2.86 (1.10)
Median (IQR)	2.5 (2.5–2.5)	2.5 (2.5–2.5)	2.5 (2.5–2.5)
Dose category, n (%)			
<2.5 mg	16 (1.9)	14 (2.7)	2 (0.6)
2.5 mg	738 (89.0)	468 (90.0)	270 (87.4)
>2.5 mg	75 (9.0)	38 (7.3)	37 (12.0)

Table S5. Baseline characteristics of all patients and patients in the subset with ≥90 days of follow-up. Abbreviations: SD, standard deviation; and IQR, interquartile range.

	**Overall (N = 829)**	**Patients with ≥90 days follow-up (N = 424)**	**Patients with vericiguat 2.5 mg as a starting dose (N = 382)**
Age (years)			
Mean ± SD	75.5 ± 11.8	73.9 ± 12.0	74.0 ± 11.9
Median (IQR)	77 (69–84)	76 (67–82)	76 (68–82)
Gender, male, n (%)	572 (69.0)	294 (69.3)	266 (69.6)
Body mass index, kg/m^2			
Median (IQR)	22.1 (19.7–24.9)	22.3 (20.3–25.0)	22.3 (20.3–25.2)
Missing, n (%)	237 (28.6)	137 (32.3)	115 (30.1)
Comorbidity, n (%)			
Hypertension	760 (91.7)	398 (93.9)	359 (94.0)
Coronary artery disease	591 (71.3)	312 (73.6)	282 (73.8)
Diabetes mellitus	498 (60.1)	259 (61.1)	237 (62.0)
Myocardial infarction	288 (34.7)	148 (34.9)	137 (35.9)
Atrial fibrillation	272 (32.8)	136 (32.1)	118 (30.9)
Stroke	138 (16.6)	71 (16.7)	60 (15.7)
Anemia	369 (44.5)	176 (41.5)	161 (42.1)
Cardiovascular procedure, n (%)			
Biventricular pacemaker	91 (11.0)	54 (12.7)	49 (12.8)
Implantable cardioverter defibrillator	451 (54.4)	66 (15.6)	58 (15.2)

Table S6. Results of univariable and multivariable Cox proportional hazard models for the factors associated with reaching the maximal daily dose of vericiguat at any given time over 90 days of treatment initiation in patients with vericiguat 2.5 mg as a starting dose. Abbreviations: SD, standard deviation; HR, hazard ratio; CI, confidence interval; ACEI, angiotensin-converting enzyme inhibitor; ARB, angiotensin-receptor blocker; MRA, mineralocorticoid receptor antagonist; ARNI, angiotensin-receptor blocker neprilysin inhibitor; SGLT2i, sodium-glucose cotransporter-2 inhibitor; GDMT, guideline-directed medical therapy, HF, heart failure. * Assessed using all available data prior to the index date. ** Assessed during 183 days prior to the index date (not included). *** GDMT included beta-blockers, ACEI, ARB, ARNI, MRA, and SGLT2i.

	**Univariate HR**		**Multivariable HR**	
	**HR (95% CI)**	**P-value**	**HR (95% CI)**	**P-value**
Age, years				
<75	Reference	Reference	Reference	Reference
≥75	0.95 (0.66–1.35)	0.772	1.01 (0.70–1.45)	0.977
Gender				
Male	Reference	Reference	Reference	Reference
Female	0.65 (0.43–0.99)	0.046	0.70 (0.45–1.07)	0.096
Vericiguat initiation setting				
Inpatient	Reference	Reference	Reference	Reference
Outpatient	1.58 (1.11–2.25)	0.012	1.56 (1.09–2.22)	0.015
*Comorbidities**				
CKD	0.93 (0.63–1.36)	0.699	–	–
Hyperkalemia	0.88 (0.58–1.33)	0.531	–	–
Anemia	0.81 (0.56–1.17)	0.256	–	–
Atrial fibrillation	1.08 (0.74–1.59)	0.674	–	–
Hypertension	2.96 (0.94–9.31)	0.063	–	–
*Device and procedure history**				
Biventricular pacemaker	0.96 (0.56–1.65)	0.881	–	–
Implantable cardioverter defibrillator	0.77 (0.45–1.33)	0.352	–	–
*HF medications***				
ACEI or ARB	1.11 (0.78–1.58)	0.563	–	–
Beta-blockers	1.58 (0.96–2.61)	0.072	–	–
MRA	1.10 (0.76–1.59)	0.628	–	–
ARNI	1.78 (1.23–2.58)	0.002	1.59 (1.08–2.35)	0.019
SGLT2i	1.57 (1.07–2.29)	0.021	1.28 (0.85–1.92)	0.237
Inotrope	0.92 (0.64–1.33)	0.668	–	–
*Number of GDMT****				
No use	Reference	Reference	Reference	Reference
Mono therapy	<0.001 (0–∞)	0.993	–	–
Dual therapy	1.53 (0.57–4.06)	0.398	–	–
Triple therapy	1.55 (0.61–3.92)	0.353	–	–
Quadruple therapy	1.74 (0.70–4.35)	0.234	–	–

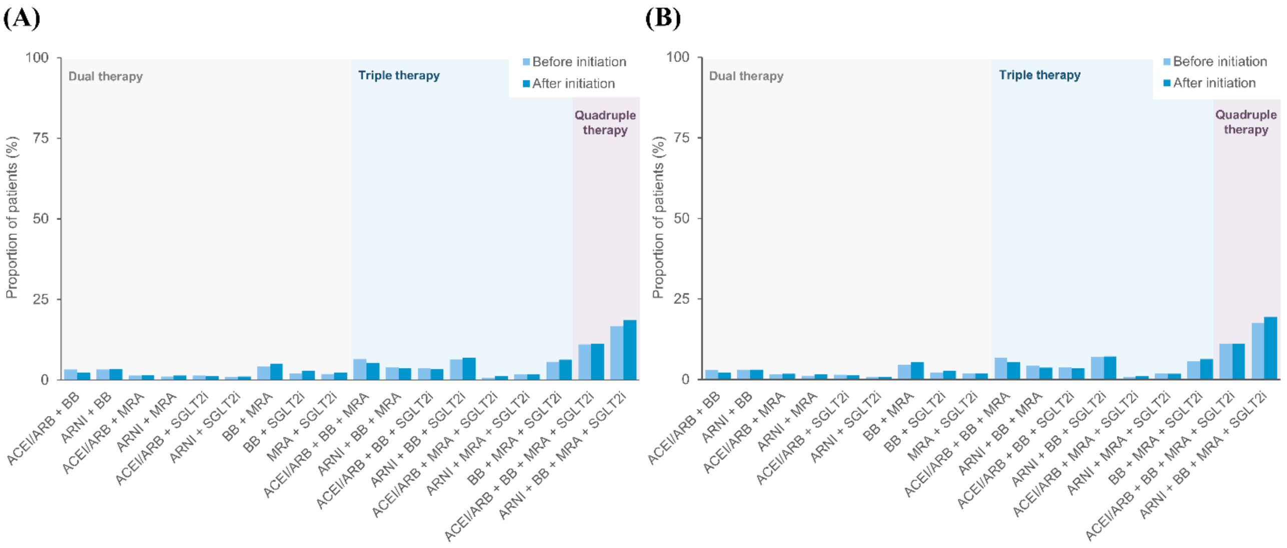

Fig. S1. Patterns of guideline-directed medical therapy combinations for heart failure before and after vericiguat initiation. Panel (A) shows the patterns of guideline-directed medical therapy combinations for HF before and after 90 days of vericiguat initiation in all patients (n = 829) and panel (B) shows those in patients with vericiguat 2.5 mg as a starting dose (n = 738). Abbreviations: HF, heart failure; ACEI, angiotensin-converting-enzyme inhibitor; ARB, angiotensin-receptor blocker; BB, beta-blockers; MRA, mineralocorticoid receptor antagonist; ARNI, angiotensin-receptor blocker neprilysin inhibitor; SGLT2i, sodium-glucose cotransporter-2 inhibitor.

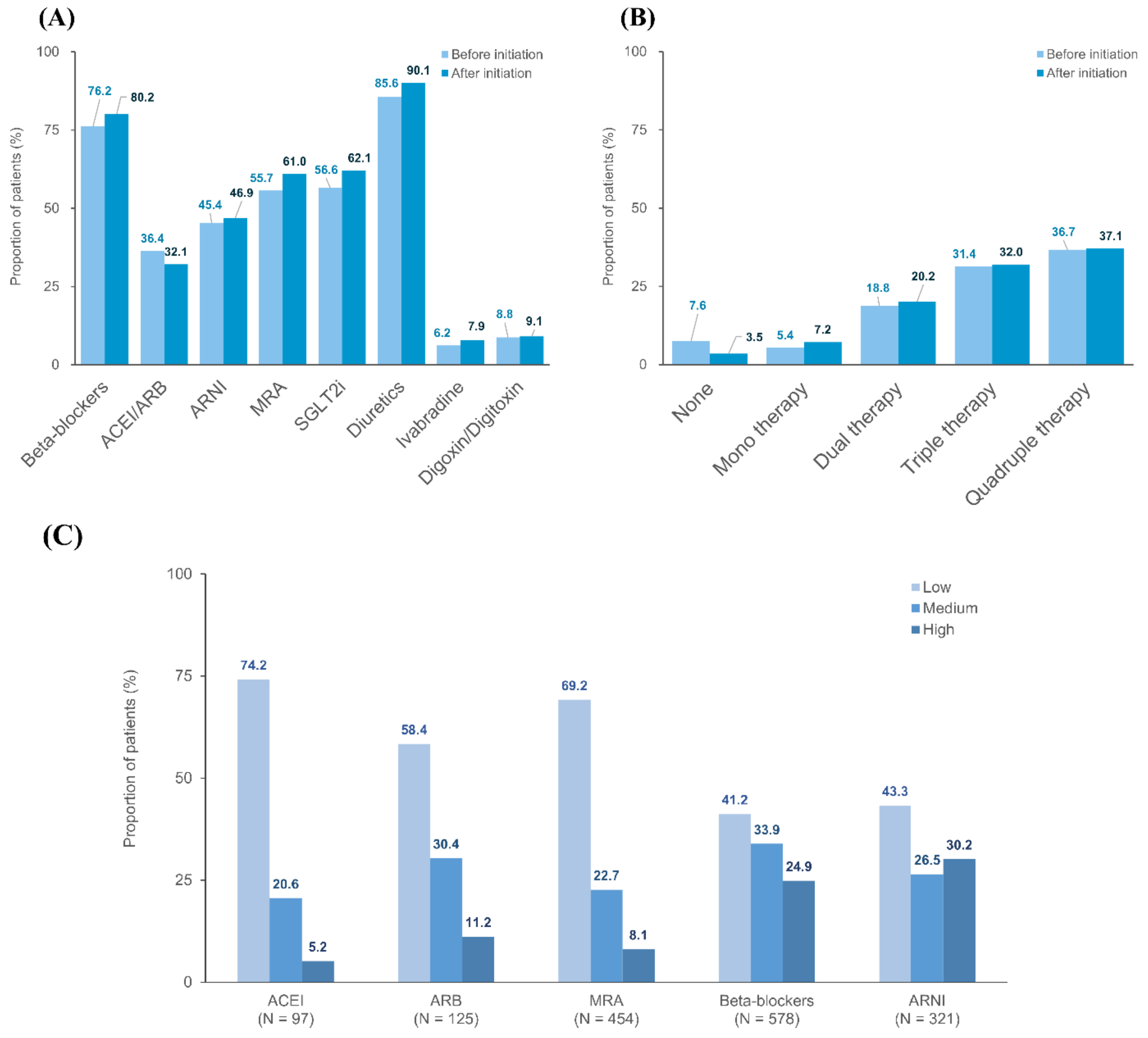

Fig. S2. Utilization patterns of heart failure medications before and after vericiguat initiation in patients with vericiguat 2.5 mg as a starting dose. Panel (A) shows the drug utilization patterns for each HF medication class before and after 90 days of vericiguat initiation; panel (B) shows the numbers of guideline-directed medicatl therapy for HF before and after 90 days of vericiguat initiation; and panel (C) shows the distribution of dosage category of each HF medication class at vericiguat initiation. The analysis was performed in patients with vericiguat 2.5 mg as a starting dose (n = 738). Abbreviations: HF, heart failure; ACEI, angiotensin-converting-enzyme inhibitor; ARB, angiotensin-receptor blocker; MRA, mineralocorticoid receptor antagonist; ARNI, angiotensin-receptor blocker neprilysin inhibitor; SGLT2i, sodium-glucose cotransporter-2 inhibitor.

References

1. Mitani H, Funakubo M, Sato N, Murayama H, Rached RA, Matsui N, et al. In-hospital resource utilization, worsening heart failure, and factors associated with length of hospital stay in patients with hospitalized heart failure: A Japanese database cohort study. *J Cariol.* 2020; **76**(4): 342-349.

2. von Elm E, Altman DG, Egger M, Pocock SJ, Gøtzsche PC, Vandenbroucke JP, et al. The Strengthening the Reporting of Observational Studies in Epidemiology (STROBE) Statement: guidelines for reporting observational studies. *Ann Int Med.* 2008; **148**(2): 573-577.
